# Systems Modeling to Quantify Safety Risks in Early Drug Development: Using Bifurcation Analysis and Agent-Based Modeling as Examples

**DOI:** 10.1208/s12248-021-00580-2

**Published:** 2021-05-20

**Authors:** Carmen Pin, Teresa Collins, Megan Gibbs, Holly Kimko

**Affiliations:** 1 Clinical Pharmacology and Quantitative Pharmacology, Clinical Pharmacology and Safety Sciences, R&D, AstraZeneca, Cambridge Science Park, Milton Road, Cambridge, UK; 2 Clinical Pharmacology and Quantitative Pharmacology, Clinical Pharmacology and Safety Sciences, R&D, AstraZeneca, Gaithersburg, Maryland USA

**Keywords:** agent-based modeling, bifurcation analysis, quantitative systems pharmacology, quantitative systems toxicology, systems modeling

## Abstract

Quantitative Systems Toxicology (QST) models, recapitulating pharmacokinetics and mechanism of action together with the organic response at multiple levels of biological organization, can provide predictions on the magnitude of injury and recovery dynamics to support study design and decision-making during drug development. Here, we highlight the application of QST models to predict toxicities of cancer treatments, such as cytopenia(s) and gastrointestinal adverse effects, where narrow therapeutic indexes need to be actively managed. The importance of bifurcation analysis is demonstrated in QST models of hematologic toxicity to understand how different regions of the parameter space generate different behaviors following cancer treatment, which results in asymptotically stable predictions, yet highly irregular for specific schedules, or oscillating predictions of blood cell levels. In addition, an agent-based model of the intestinal crypt was used to simulate how the spatial location of the injury within the crypt affects the villus disruption severity. We discuss the value of QST modeling approaches to support drug development and how they align with technological advances impacting trial design including patient selection, dose/regimen selection, and ultimately patient safety.

## INTRODUCTION

Quantitative methods are well established across many therapeutic areas to evaluate the safety and efficacy of an investigational drug during development (1). In the past 10 years, this field has seen an unprecedented development with the emergence of Quantitative Systems Pharmacology/Toxicology (QSP/T) models. QSP/T models have arisen from the integration of pharmacokinetics (PK) with systems biology approaches to enable the quantification of dynamic interactions between drug(s) and biological processes, intended or unintended, in the organism as a whole (2). In a similar fashion to physiologically based pharmacokinetics (PBPK) modeling, which is now widely accepted as a tool for regulatory decision-making, QSP/T modeling is growing in use for regulatory submissions and responses (3).

QSP/T modeling aims at merging the drug disposition kinetics in plasma, tissues, and cells with the multiscale nature of interplaying organic systems and thus lead to a paradigm shift from scale-specific reductionistic approaches to multiscale models (4,5). This paradigm shift is further endorsed by the increasingly available wealth of information within complex data of multiscale nature that spans levels of organization from cells to whole organisms. Multiscale models can provide predictions of the system’s behavior under pharmacological and toxicological challenges at each structural level as well as a whole and, through scale bridging methods, transfer information between scales (6).

Importantly, QSP/T modeling facilitates the increasing integration of complex experimental *in vitro* techniques, including organoids and microphysiological systems, into the pharmaceutical testing strategy (7–14). QSP/T models and *in vitro* systems are entangled in a mutually supportive relationship. The information derived from *in vitro* systems may not have a straightforward interpretation without a mathematical framework that can account for differences in exposure profiles and physiological changes over time (15). *In vitro* systems provide a variety of dynamic, high-resolution measurements of multiscale nature and can be enhanced with cell lineage tracing techniques, live imaging, genetic engineering, multi-drug screening, and co-culture methods, which all together advance our understanding of pharmacological and toxicological systems. As a result, the opportunity emerges to leverage mechanistic models able to bridge *in vitro* endpoints with clinical responses in preclinical stages during drug development.

One of the main applications of QSP/T models lies at the translational interface between the identification of a candidate drug and its use in clinical trials to address, in particular, safety risks mediated by on-target mechanisms. On-target toxicities cannot be simply screened out because of their intrinsic relationship with efficacy while their evaluation and management during clinical studies can be extremely costly. In such instances, therapeutic margins can be rationally predicted by applying QST modeling approaches in preclinical stages, thereby increasing the clinical probability of success. QST models enable the quantification of the dynamics of toxicity and recovery of drug-affected organs and, thus, they can facilitate decisions of effective and tolerated dose schedules.

In the following sections, we examine the application of QST models in the development of drugs exhibiting hematological and gastrointestinal adverse effects (AEs). Such compounds include chemotherapy, antibody-drug conjugates (ADCs) with cytotoxic payloads and immunotherapy, and targeted therapies involving the cell cycle and DNA damage response and repair mechanisms. We explore two QST modeling approaches used to quantitively assess these toxicities and conduct simulations to reveal particular phenomena which may need attention during drug development.

## MODELING HEMATOLOGICAL TOXICITY OF ONCOLOGY THERAPIES

The most common cause of blood cytopenias in the industrialized world today is myelosuppression occurring as an adverse effect of cytotoxic chemotherapy for malignant neoplastic disease. Drugs in this category typically produce dose-dependent myelosuppression, which reverts after treatment discontinuity (16). The production of all mature blood cell types results from the concerted action of hematopoietic stem cells (HSC) and progenitor cells in the bone marrow. Cell proliferation and differentiation are tightly regulated to give rise to specific numbers of all blood cell types (17). Furthermore, hematopoietic homeostasis is maintained by the complex interplay between extrinsic cues and intrinsic regulatory pathways, including cytokine-mediated feedback loops between mature cells and progenitors (18,19).

Since 2000s, the area of chemotherapy-induced hematotoxicity has seen QST modeling approaches, integrating PK profiles with cell dynamics models (see (20) and (21) for reviews on this topic). A well-known approach is the model of chemotherapy-induced myelosuppression published by Friberg and co-workers in 2002, which comprises a system of nonlinear ODEs to account for a pool of proliferating cells, a set of transit nonproliferating cell compartments, a pool of circulating mature cells, and a negative feedback loop between mature cells and their progenitors (22). Modified versions of this model with increased granularity of the feedback regulatory mechanisms have been proposed to improve predictive performance (23,24). These models are frequently applied to quantify hematotoxicity of complex dose schedules in patients (25–27) as well as in preclinical settings (28).

Considering a single chain of compartments to describe toxicity affecting one of the several blood cell types may not be always sufficient to describe observed behaviors. For instance, erythropoietin increases in response to low red blood cell counts and stimulates, in turn, erythroid progenitor proliferation but also impacts on platelet production (29). Describing such cross-lineage feedback regulation requires more complex network structures. An example of a QST model considering the hematopoietic tree with interacting multiple lineages and common progenitors responding to feedback loops has been developed to quantify the dynamics of several cytopenias associated with carboplatin treatment in preclinical settings (30). This model highlights the complexity of the feedback mechanisms acting upon both proliferation and differentiation and the relevance of common feedback regulation of different cell progenitors to understand the dynamics of the hematopoietic system as a whole during toxicity and recovery (30). Similarly, a recent study focused on the development of a QST model of *in vitro* hematotoxicity data from a high-throughput novel multi-lineage toxicity assay has been used to characterize the hematological profile of several multi-class anti-cancer agents and to investigate the mechanisms of toxicity (31). The spatial organization of the bone marrow has also been considered by a multiscale *in silico* model, which incorporates three distinct spatial scales—cell, hematopoietic subunit, and bone marrow (32). This model has provided a plausible explanation for the emergence of the fractal-like spatial organization of bone marrow trabeculae and sinuses as the result of maximizing mature cell production within the volumetric restrictions of the bone marrow.

Most of the models of the hematopoietic system include feedback regulatory loops, which greatly influence the qualitative or topological structure of the models. Several studies have highlighted the importance of performing stability and bifurcation analysis to understand the model behavior and its dependence on parameter values (33,34). These studies have reported a Hopf bifurcation, which is a change in model behavior from non-oscillatory to oscillatory, associated with the parameter(s) regulating the feedback between mature cells and bone marrow progenitors in the classical and modified Friberg models (33,34). In the classical model (22), changes in the qualitative behavior are determined by the values of the parameter *γ*, which describes the strength of the feedback regulation between mature cells and progenitors (34). With *γ* values smaller than approximately 0.5685, the homeostatic blood cell concentration represents an asymptotically stable equilibrium in the classical Friberg model (34). Thus, after perturbations, such as cytotoxic drug challenges, the predicted cell concentration eventually recovers to the basal/homeostatic value. With larger values of *γ*, the predicted concentration does not return to a constant value but keeps oscillating around the basal value with cycles of stable magnitude (34). The latter behavior is not representative of reversible chemotherapy-induced cytopenia, and hence, values of the parameter *γ* should be restricted to values smaller than 0.5685 when modeling this scenario. However, even values of *γ* ensuring the return to constant, non-cyclic, homeostatic blood counts can result in non-physiological predictions due to the magnitude of oscillations and/or time to return to equilibrium.

**Fig. 1:**
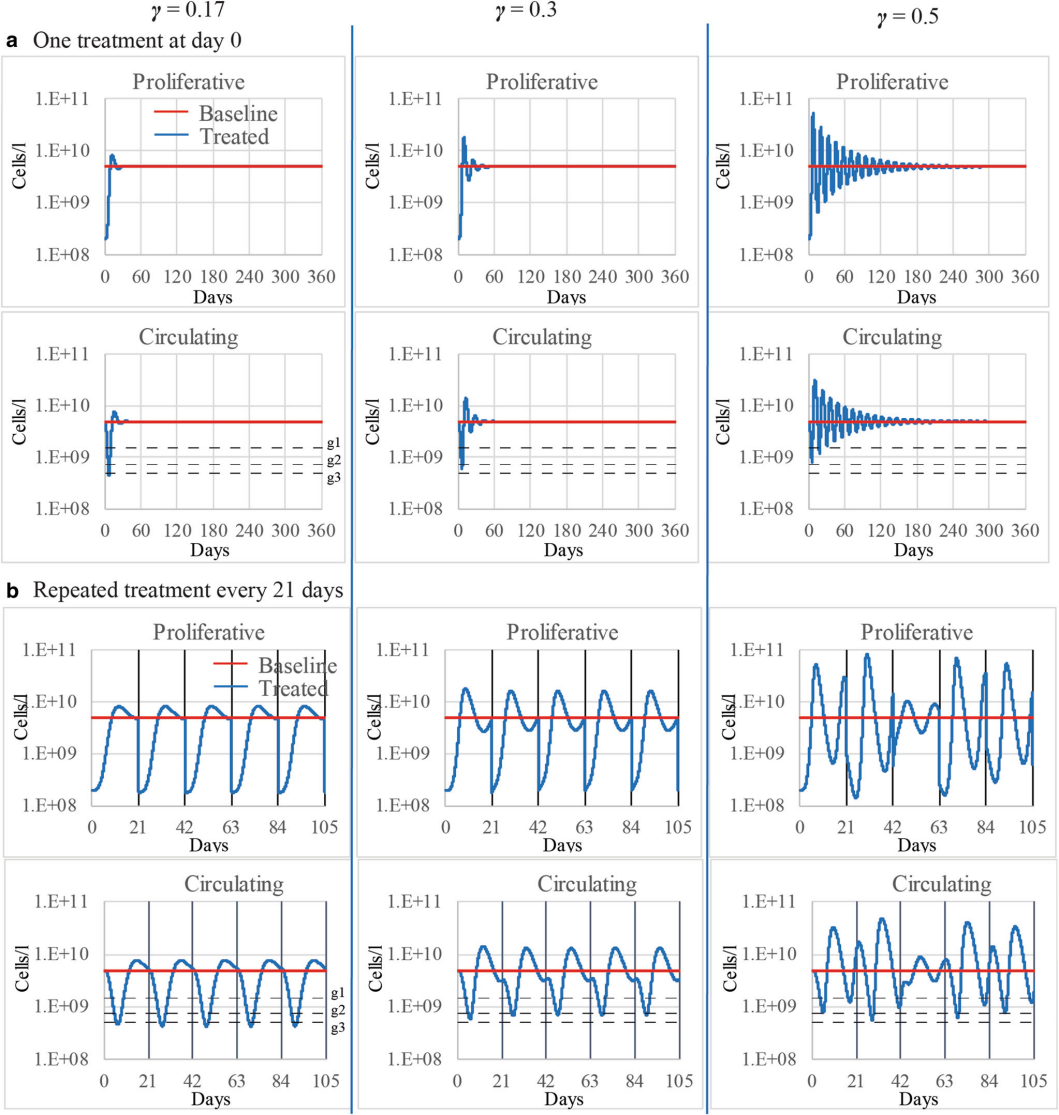
Impact of the parameter *γ*, i.e., strength of the feedback between mature cells and proliferative progenitors, on the qualitative behavior of the classical Friberg model (22) of neutrophil dynamics following drug insult. We used several values of *γ* and previously published values for the other model parameters (22). The chosen values of *γ* ensured stable non-cyclic neutrophil blood counts in homeostasis and return to baseline value after drug treatment discontinuity (*γ* < 0.5685). **a** Simulation results of neutrophil and progenitor recovery after a single treatment killing 95% of proliferative progenitors indicated that high values of *γ* resulted in long periods of non-physiological large oscillations of neutrophils and progenitors before returning to constant homeostatic levels. **b** Multiple treatments killing 95% of proliferative progenitors every 21 days resulted in large feedback-driven oscillations of neutrophils and progenitors with highly irregular profiles and no return to homeostatic values in between treatments for the largest value of *γ.* Dashed lines represent limit values defining the common terminology criteria for adverse effects (grade 1, 2, or 3 neutropenia)

To investigate the model behavior in more depth, we have simulated neutrophil dynamics during a theoretical treatment that kills 95% of proliferative progenitors and the subsequent recovery for various values of γ which all ensured stable non-cycling neutrophil blood counts in homeostasis (*γ* < 0.5685). Injuries affecting 95% of bone marrow cells have been previously reported in *in vivo* experiments with oncotherapeutics (30). We used the previously parametrized classical Friberg model for human myelosuppression (22) with the published value *γ* = 0.17 and tested two values of greater magnitude, 0.3 and 0.5. We observed that for γ = 0.17 and γ = 0.3, neutrophil homeostasis is recovered in less than a month after a single treatment. However, *γ* = 0.5 leads to oscillating concentrations of neutrophils in blood with progressively smaller cycles and it requires months to recover the constant basal value after a single treatment (Fig. 1a). On a different scenario, we simulated repeated treatments every 21 days and observed that values of 0.17 and 0.3 result in consistent decreases of neutrophils in response to treatment administrations (Fig. 1b). However, for values of 0.5, we observed highly irregular neutrophil profiles in which variations associated with repeated administrations were masked by large feedback-driven oscillations of mature cells and progenitors (Fig. 1b). With our simulations, we have demonstrated the usefulness of bifurcation analysis to reveal changes in the model qualitative behavior associated with changes in the value of γ. From a practical perspective, while data quality and resolution are often sufficient to prevent estimated values of γ that result in physiologically implausible oscillations, it is important to acquire a full understanding of the relationship between the parameter space and the model qualitative behavior.

As highlighted above, the classical and modified versions of the Friberg model can describe cyclic oscillations or stable blood cell counts depending on the value of the parameter governing the feedback regulation between mature cells and progenitors (33,34). Models including a mechanistic description of this feedback regulation (35,36) could result in easier interpretation of parameters and detection of non-physiological behaviors. However, the complexity derived from the implementation of this feedback regulation, either in empirical or in more mechanistic approaches, results in nonlinear terms in the system of differential equations that require the assessment of the model qualitative behavior to ensure that it agrees with our understanding of the process. While sensitivity analysis reveals the impact of input parameters on the modeled output quantities and may facilitate the identification of bifurcation parameters, it does not provide any information on the topological structure of the model and how this changes across the parameter space. Despite the difficulty to compute stability boundaries of equilibria, find limit cycles, or predict qualitative changes in the system behavior, bifurcation analysis can enhance the development and use of mechanistic models to describe nonlinear effects, which are frequently encountered in pharmacological process.

## MODELING GASTROINTESTINAL TOXICITY OF ONCOLOGY THERAPIES

Gastrointestinal (GI) side-effects of chemotherapy present a constant challenge in the efficient and tolerable treatment of cancer and are among the primary reasons for dose reductions, delays, and cessation of treatment. The incidence of chemotherapy-induced diarrhea has been estimated to be as high as 80% (37). It is widely believed that chemotherapy-induced GI dysfunction revolves mainly around mucosal damage and ulceration, which is initiated by the direct or indirect effects of cytotoxic chemotherapeutics on the rapidly dividing epithelial cells in the GI tract (37).

The intestinal epithelium comprises a cellular monolayer folded to form invaginations or crypts and protrusions or villi in the small intestine. The epithelium undergoes continuous cell renewal driven by stem cells located at the base of the crypts (38). Stem cells proliferate and their progeny migrate upwards while proliferating and differentiating into one of several epithelial functional types (39). The equilibrium of this dynamical system is maintained by compensatory cell shedding into the gut lumen (39). Several modeling approaches have been proposed to describe the complexity and dynamical nature of the epithelium. Compartment models with variable granularity have been used to quantify the temporal dynamics of epithelial cell types (40–45). A complete description of epithelial dynamics is required to capture the well-understood spatial cell dynamics within crypts and villi. The spatial dimension has been considered in cell population models using partial differential equations (46) as well as in agent-based models that are computational models implementing individual cell behaviors and interactions using two- or three-dimensional spatial representations of the crypt-villus structure (47–50).

QSP/T models integrating PK profiles with epithelial dynamics are instrumental to precisely quantify the disruption in cell proliferation and recovery following anti-cancer therapies. Most of the analytical and computational models developed to answer basic epithelial biology questions are applicable to understand the disturbance of the epithelium when exposed to dynamical drug concentration profiles. An example of an analytical model has been developed to describe the disturbance of epithelial homeostasis during irinotecan exposure and its recovery after drug clearance (51). This model describes the temporal dynamics of stem cells, daughter cells, and enterocytes using a system of ordinary differential equations. It is calibrated for humans and rats to enable translation of toxicity and provides a meaningful relationship between enterocyte loss and GI AE in patients.

The high performance of computational models has been demonstrated in spatiotemporal simulations of colon cancer initiation and treatment (52) and intestinal tumorigenesis triggered by Wnt-activating mutations (53). Computational models are amenable to develop multiscale approaches that integrate processes spanning multiple temporal and structural levels of organization (e.g., molecules, cells, organ, organism). For instance, computational models comprising molecular processes governing individual cell fate have been applied to simulate cell dynamics in aging crypts (54) and to understand the spatial relationship of molecular signals and growth patterns (55). The development of these models is greatly facilitated by novel microphysiological systems, which enable experimental designs unfeasible in other experimental platforms, and hence can help reveal unknown aspects of GI biology and toxicity (8).

It is noteworthy that computational models can describe single cell dynamics within the three-dimensional spatial configuration of the crypt. In this regard, proliferation-driven forces, which determine cell migration across crypts and villi, leading to epithelial turnover, vary according to the cell position within the crypt geometry (43,46,56). In addition, computational models enable the simulation of biological events that are not tractable or straightforward to describe analytically. For instance, crypt extinction, crypt fission (57,58), cell plasticity, and dedifferentiation (59) are all well-described phenomena observed during epithelium disruption and regeneration, which can be implemented in computational models we used below in our simulations. Agent-based modeling enables the exploration of dynamics that are out of the reach of analytical approaches by relying on computing power.

**Fig. 2:**
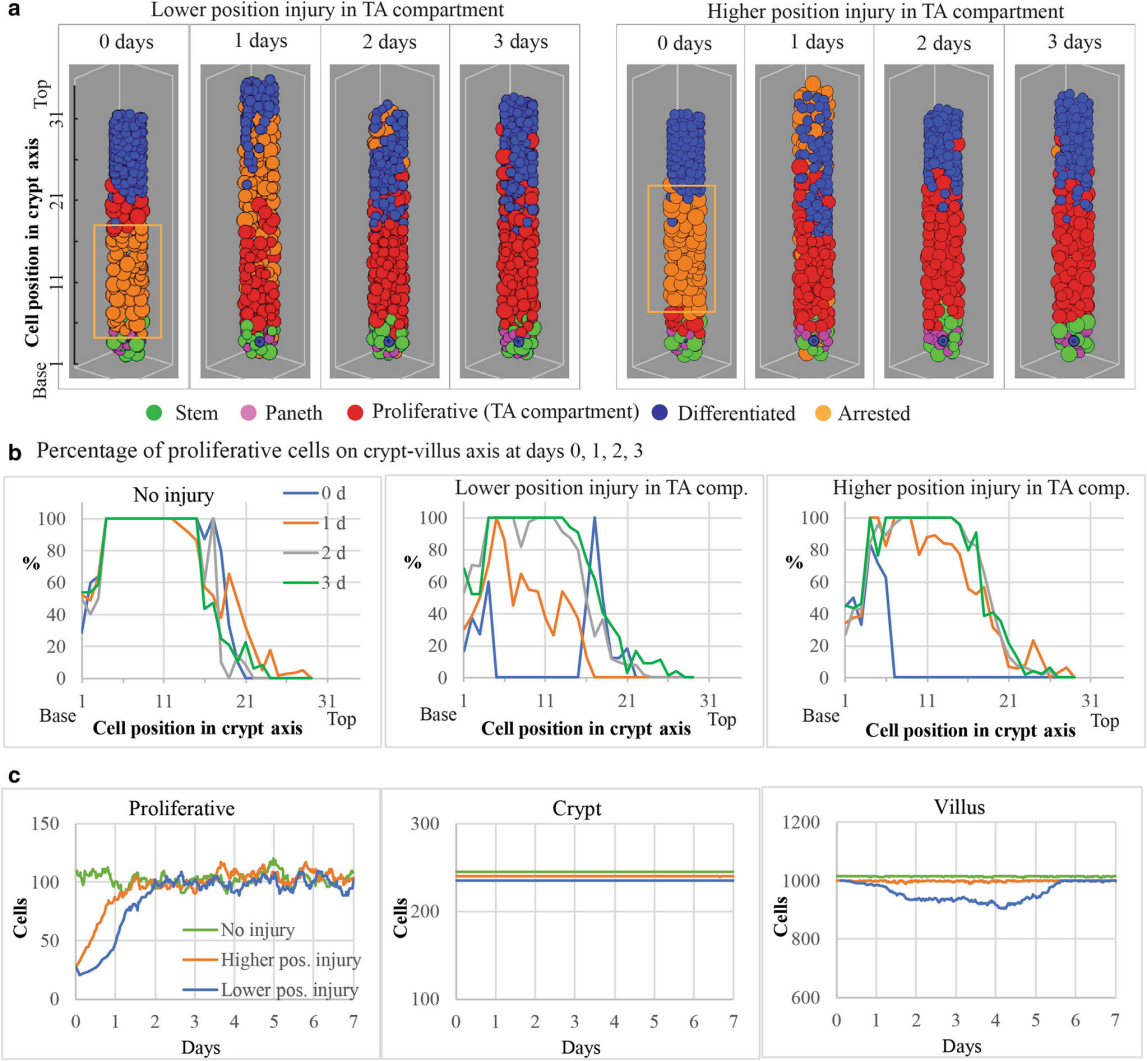
Impact of the crypt spatial configuration on simulated dynamics of epithelial injury and recovery. **a** Cartoon representing snapshots of an agent based model of a mouse small intestinal crypt recovering after induction of cell cycle arrest in 85% proliferative cells located at low and high positions (from the base) of the transit amplifying (TA) compartment. Recovery is achieved by proliferation of non-injured cells. Boxes mark affected crypt areas. **b** The spatiotemporal simulation of the percentage of proliferative cells in the crypt shows that cells located between approximate positions 5 and 20 in the crypt axis are mostly proliferative progenitors forming the TA compartment in healthy intestine. Simulation of the injuries described in **a** showed that the recovery of the TA compartment required more than 2 days following the arrest of 85% cells at lower positions but shorter time if the same injury was located at the higher position of the TA compartment. **c** The simulated number of cells over time after the injuries described in **a** indicated that the total number of cells in the crypt is not affected in these scenarios because arrested cells are replaced by newly generated cells within the crypt before the onset of cell senescence. However, the number of cells in the simulated villus is compromised for a relatively long period following the injury at lower position but not affected when the same injury was located at higher position in the crypt

To demonstrate the usefulness of agent-based models, we performed a simulation exercise with hypothetical scenarios in which spatial cell dynamics within the crypt geometry to describe epithelial injury and recovery was accounted for. Using the previously developed agent-based model of the mouse small intestinal crypt (50), we simulated the induction of cell cycle arrest in approximately 85% of proliferative cells located at low or high positions of the crypt transit amplifying compartment (Fig. 2a) and followed post-perturbation cell dynamics in the crypt and villus. *In vivo* experiments show reductions equal to or larger than 85% of the crypt proliferative activity associated with cancer treatments (60,61). The transit amplifying compartment comprises a relatively large number of proliferative cells located above the stem cell niche (Fig. 2a). We assumed that the simulated cycle arrest process was irreversible and the time to senescence of arrested cells was similar to that of absorptive epithelial cells. The recovery of the crypt was based on the remaining unaffected proliferative cells, which divided and replaced arrested cells while forcing their migration upwards and onto the villi. Our simulation results showed that the recovery of the proliferative compartment within the crypt was faster when arrested cells were located at the higher position in the crypt (Fig. 2a, b, and c left plot). This is explained by the collective dynamics, which emerges from cell proliferation within the crypt geometry and results in faster cell migration velocity driven by greater proliferation-derived forces at the higher crypt region (50,62). Thus, while higher position crypt injuries were relatively quickly resolved and did not impact on the villus structure, lower position crypt injuries resulted in reduced proliferation for longer periods, which led to decreased migration of cells into the villus and compromised the villus integrity (Fig. 2c right plot). These simulations show how a relatively simple agent-based model can predict complex behavior involving different outcomes on epithelial integrity of the villus associated with the same injury occurring at different positions in the crypt.

A well-understood injury with a complex spatial pattern in the crypt-villus axis is caused by LPS, which induces rapid TNF-mediated epithelial cell apoptosis exclusively at the villus tip (44,63). The reasons why oncology therapies may or may not be associated with GI AEs are not well understood yet. Our modeling results show that the spatial location of the injury in the crypt could be a factor determining the emergence of clinical AE. Proving this hypothesis is challenging because measurements with suitable spatial and temporal resolution are rarely available. However, given the spatial complexity in cell composition and signaling pathways across the crypt-villus axis, there is a credible possibility that drug interventions could affect only specific spatial regions where responsive cells or molecules are located. For such cases, models capturing the spatiotemporal dynamics of single cells and molecules are instrumental to quantify the response, test hypotheses, and unravel details of the toxicological process not always feasible to assess experimentally.

The highly dynamic nature of cell behaviors and interactions in the 3D crypt-villus architecture makes the epithelium a suitable system to develop agent-based models with the capacity to describe molecular processes, single cell behavior, and the emergence of collective cell dynamics. In the field of GI drug safety, where patient data acquisition is challenging and often reduced to symptom description (e.g., presence and/or severity of diarrhea), agent-based models can emulate biological mechanisms at multiple scales and may allow unanticipated behaviors to emerge and provide precise quantitative information on AEs based on the compound mechanism of action in early stages of drug development.

## CONCLUDING REMARKS

The modeling approaches we have used in our examples focus on the quantification of cell dynamics in the bone marrow and intestinal epithelium during anti-cancer treatment and recovery. Understanding how to improve safety by optimizing dose and regimen can bring in significant therapeutic benefit for oncology patients. We demonstrated the importance of bifurcation analysis in QST models of hematologic toxicity to understand how the parameter space determines different model qualitative behaviors, which may not be representative of reversible chemotherapy-induced cytopenia. In addition, using a systems model for the intestinal crypt, we simulate the effect of the spatial location of the injury within the crypt on the severity of the disruption of villus integrity. These models are readily amenable to be integrated with other relevant features spanning levels of physiological organization from molecular drivers of pharmacological efficacy to clinical adverse effects.

QSP/T models offer a framework where PK is connected to dynamical processes of efficacy and safety, with the opportunity of utilizing various inputs/outputs in therapeutic index calculations via exploration of different dose schedules. The development of novel modeling solutions is critically important in the oncology space with the focus on combination therapy and new treatment-delivery modalities (e.g., expansion of cell therapy, antisense oligonucleotides, nanopeptides, and ADCs) in development. For example, the application of ADCs delivering cytotoxic warheads in the treatment of cancers can be enhanced by the use of tailored QST models able to uncover critical determinants of therapeutic index including disposition, warhead’s cell entry pathway, and susceptibility mechanisms in on- or off- target cells (64), which will enable future selection of ADC properties. Similarly, adoptive cellular therapy is now enjoying unprecedented bench-to-bedside clinical success in the fight against cancer (65,66). Successful immunotherapy requires understanding of the dynamic interactions among intrinsic immunity, tumor environment, and the immune agent driving the therapeutic response and how this can lead to immunological adverse effects such as cytokine release syndrome. Understanding these processes will be greatly facilitated by modeling approaches specifically designed to handle such complexities (67).

QSP/T models are increasingly consolidated in the field and currently co-exist with empirical PKPD pharmacometrics approaches. The application of simple, empirical models or more complex, mechanistic approaches to drug development has been comparatively discussed by several authors (68–71). The practical choice of modeling technique is likely made by balancing the investment required to develop or apply QSP/T models and the importance of those questions that cannot be addressed by empirical approaches. The ability to combine modeling approaches to optimize therapeutic index will be of utmost importance moving forward to gain a mechanistic understanding of determinants of safety and efficacy in various patient populations.

We are witnessing a very rapid technological advancement of data science and artificial intelligence (AI) approaches in the pharmaceutical sector. These are empirical approaches that offer a range of complementary tools, which enable the identification of relationships among genes, proteins, drugs, and phenotypes using large and complex datasets (72,73). Recent AI advancements in image analysis algorithms allow training to perform tasks such as nuclear or cellular segmentation and tissue classification, and with the digital pathology image data, an agent-based modeling could be developed to characterize T cell clustering and spatial intra-tumoral heterogeneity (74). AI tools are currently regarded valuable in informing the development of advanced mechanistic QSP/T models, and we expect many reports on successful applications in the near future.

Currently, there is a growing need to interpret the increasing wealth of information that is now available in clinical trials under increasingly short timelines to impact drug development decisions. QSP/T modeling can reveal unexpected useful target pathways for drug development (75), which may be overlooked without quantitative understanding of physiology, pathology, and drug mechanism of action. However, QSP/T model development can take several months or years; therefore, the decision to apply these models must be made early with rational intention to explain empirical study results. Strategic investment in QSP/T models must be taken as a partnership between modelers, experimental scientists, clinicians, drug development project teams, and leadership from early drug development, as the ability to differentiate compounds at lead selection and in early development has the potential to decrease candidate attrition and shorten drug development cycle time.

## Contribution as Female Scientists

**Carmen Pin:** Academics and professionals are actively collaborating within the pharmaceutical sector to develop novel modelling solutions for the quantitative assessment of drug safety and efficacy. Common interests are giving rise to highly exciting and multidisciplinary working spaces, such as TransQST consortium, where advanced modelling techniques are being developed and put to the test in realistic toxicology scenarios. I feel fortunate of working in such a rewarding environment.

**Teresa Collins:** Working in a large pharmaceutical company for the last 17 years has taught me that there are a wealth of opportunities to address drug project issues using modelling and simulation, and the key issue is how best to support these from a range of modelling approaches. In particular, understanding when, what and how to invest time and effort in developing a QSP/T model. From the outset it is important to have initial conversations with potential collaborators, and decide if there is (1) sufficient data, (2) biological understanding/hypothesis, (3) clear aims, and (4) scope to impact a range of programs. If your intuition suggests you can fulfill these four criteria then you are in a strong position to turn a great idea into a valuable decision making tool within drug development.

**Megan Gibbs:** For the past 20 years a focus on the expansion of quantitative methods in Biopharma has enabled virtual trials, virtual patients and virtual systems to inform drug development decision making. Initially the development of mechanistic PKPD models to understand pharmacologic effect, modeling of safety biomarkers to determine therapeutic index, and model based meta-analysis to understand competitive advantage has now taken us to the interface of AI, systems modeling and digitization to further enhance our scientific understanding. I am humbled by the talented scientists of which I have the privilege to interact and their continued focus on impacting patients.

**Holly Kimko:** Dr. Crusher scans an injured sentinel with a medical tricorder in her sickbay to predict when he would become conscious to report to Captain Picard. More than 30 years ago, this scene of Star Trek sparked my interest in the development of such a device, which must include an algorithm able to predict changes in physiology based on the simultaneous detection of multiple biomarkers. I became a pharmacometrician, supporting mainly phase 2/3/4 clinical drug development by pharmacokinetic/dynamic modeling & simulation to characterize clinical responses that reflect, only at a high-level generality, enormous complexity intertwined among biomarkers at molecular, cellular, tissue/organ-level changes. Extending the modeling approach, systems modelling connects biomarkers spanning multiple scales to predict non-clinical and clinical responses to candidate drug interventions, and it has advanced rapidly during the last decade to enable a more efficient exploration of the drug development universe. Now, I know that I am inching towards the realization of the scene in the sci-fi movie. Engage!
